# Origin and Mechanism of Piezoelectric and Photovoltaic Effects in (111) Polar Orientated NiO Films

**DOI:** 10.1002/advs.202304637

**Published:** 2023-09-26

**Authors:** Nana Fan, Yingfei Wang, Bin Liu, Heqing Yang, Shengzhong Liu

**Affiliations:** ^1^ Shaanxi Key Laboratory for Advanced Energy Devices Shaanxi Engineering Laboratory for Advanced Energy Technology Key Laboratory of Macromolecular Science of Shaanxi Province School of Materials Science and Engineering Shaanxi Normal University Xi'an 710119 China; ^2^ Key Laboratory of Applied Surface and Colloid Chemistry National Ministry of Education Shaanxi Key Laboratory for Advanced Energy Devices Shaanxi Engineering Lab for Advanced Energy Technology School of Materials Science and Engineering Shaanxi Normal University Xi'an 710119 China

**Keywords:** NiO thin film, [111] orientation, origin and mechanism, photovoltaic effect, piezoelectric effect, polar structure

## Abstract

Based on the current piezoelectric theory, NiO with the centrosymmetric structure is not piezoelectric. However, herein, this study shows the first observation of piezoelectric generation, rectifyingand bulk photovoltaic behaviors in NiO films with [111] orientation and the change in NiO crystal structure in piezoelectric process. The piezoelectric generation, rectifying, and bulk photovoltaic performances are enhanced by increasing (111) orientation, and attenuated and eliminated by applying a persistent stress on the NiO film. The NiO [111] is polar direction, and thus a spontaneous electric field (E_S_) is in the NiO film with [111] orientation. The existence of Es in (111) oriented NiO film is found to be the physical basis of the piezoelectric generators and photovoltaic and rectifying effects. Thus, NiO piezoelectric, rectifying, and bulk photovoltaic mechanism are presented at the atomic level. The mechanism may rewrite the current piezoelectric theory, and establish a unified theory of polar structure with wide implications. The polar‐orientated films can be used to fabricate piezoelectric generators and other optoelectronic devices with high performances.

## Graphical abstract

Based on the existing piezoelectric theory, NiO with the centrosymmetric structure is not piezoelectric. However, herein, this study shows the observation of piezoelectric, rectifying and photovoltaic effects in (111)‐oriented NiO films. These effects are attested to originate from the electric field produced by spontaneous polarization in the (111)‐oriented NiO film. The polar structure piezoelectric and photovoltaic mechanism is thus presented.

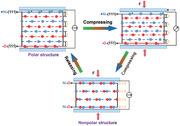

## 1 Introduction

Since the piezoelectric effect was observed in quartz crystal by the brothers Curie in 1880 for the first time,^[^ ^1^ ^]^ the piezoelectric materials and devices have attracted much attention owing to their applications in sensors, energy harvesters, ultrasonic motors, imaging devices, multilayer actuators, and so on.^[^ ^2^, ^3^, ^4^, ^5^, ^6^ ^]^ For a long time, the piezoelectric effect has been considered that piezoelectric materials are polarized under an applied external stress, and the polarization is proportional to the applied stress.^[^ ^7^ ^]^ The piezoelectric effect is observed only in non‐centrosymmetric crystals.^[^ ^8^ ^]^ Whereas, the centrosymmetric crystals are not piezoelectric.^[^ ^9^ ^]^

The first ZnO nanowire array nanogenerator was demonstrated by Wang et al. in 2006.^[^ ^10^ ^]^ Subsequently, various piezoelectric nanogenerators based on ZnO nanowires/nanorods^[^ ^11^, ^12^, ^13^, ^14^, ^15^, ^16^, ^17^, ^18^, ^19^, ^20^, ^21^, ^22^, ^23^, ^24^, ^25^, ^26^ ^]^ and thin films^[^ ^27^, ^28^ ^]^ have been developed. In addition, the piezoelectric nanogenerators based on CdS nanowires,^[^ ^29^, ^30^ ^]^ CdTe micro/nanowires,^[^ ^31^ ^]^ GaN nanowires,^[^ ^32^, ^33^, ^34^ ^]^ InN nanowires,^[^ ^35^ ^]^ ZnSnO_3_ micron belt,^[^ ^36^ ^]^ ZnS nanowire arrays,^[^ ^37^ ^]^ ZnO/ZnS heterogeneous nanowire arrays,^[^ ^37^ ^]^ polytetrafluoro‐ethylene nanofibers,^[^ ^38^, ^39^ ^]^ and PbTiO_3_ nanowire arrays^[^ ^40^ ^]^ have been reported. A piezoelectric potential(piezopotential) was generated in a micro/nano scale 1D structure through dynamic straining. The existence of piezopotential is the physical basis of these nanogenerators.^[^ ^41^ ^]^ When a strained micro/nano scale structure is linked to an external load, the piezopotential drives electrons in the circuit to flow. The mechanical energy is thus converted into electric energy.^[^ ^41^, ^42^ ^]^ However, based on this model, it is difficult to understand the formation of piezoelectric pulse current and the orientation‐dependent piezoelectric generation properties.

NiO is a very important p‐type semiconductor, it has been widely studied owing to its potential application in photocatalysis,^[^ ^43^ ^]^supercapacitors,^[^ ^44^ ^]^ lithium‐ion batteries,^[^ ^45^ ^]^ magnetic materials,^[^ ^46^ ^]^ nonvolatile memory,^[^ ^47^ ^]^ and so on. The NaCl‐type structure NiO contains a symmetrical center. Based on the current piezoelectric theory, NiO is not piezoelectric.

Recently, we observed the [001] polar orientation enhanced piezoelectric generation performances in ZnO films.^[^ ^48^ ^]^ The piezoelectric effect is evidenced to originate from the spontaneous polarization in the [001] polar direction of ZnO, and thus a novel piezoelectric atomic mechanism is presented.^[^ ^48^, ^49^ ^]^ Furthermore, we found the NiO polar {111} facet enhanced photocatalytic activity and presented a model of charge separation between polar NiO {111} facets.^[^ ^50^ ^]^ To determine the origin of the piezoelectric effect, herein, we show the first observation of piezoelectric, rectifying, and photovoltaic effects in NiO films with [111] orientation and the change in NiO crystal structure in piezoelectric process. The spontaneous electric field (Es) generated by the spontaneous polarization in the NiO film is found to be the origin of the rectification, piezoelectric, and photovoltaic effects, and the corresponding physical mechanism at the atomic level is presented. This work establishes the polar structure piezoelectric and photovoltaic mechanism and may rewrite the current piezoelectric theory.

## 2 Results and Discussion

### 2.1 NiO Films with Different [111] Orientations

**Figure 1:**
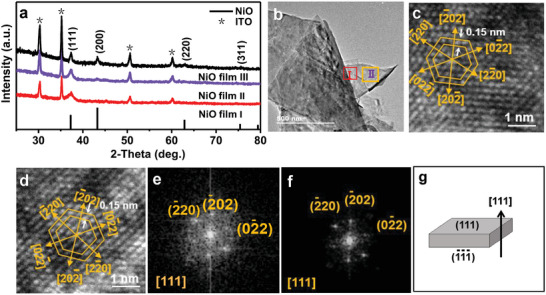
a) XRD patterns of the NiO films I, II and III obtained by changing the volume ratio of ethylene glycol to water. The stick pattern in (a) is the standard XRD pattern of NiO powders with Cu K_α1_ radiation (Joint Committee on Powder Diffraction Standards card file no.47‐1049). b) Typical TEM image of NiO film І. c,d) HRTEM images from boxes І and II, respectively. e,f) Corresponding FFT patterns from (c) and (d), respectively. g) Crystal orientation illustration of NiO film with an unitary (111) orientation.

NiO films with different texturing coefficients of (111) were obtained by adjusting the volume ratio of ethylene glycol(HOCH_2_‐CH_2_OH) to water(H_2_O) in the preparation of the NiO precursor gel. When the volume ratio of HOCH_2_‐CH_2_OH to H_2_O is 2:1, 1:13, 0:14, the as‐obtained NiO film was labeled as NiO film I, NiO film II, and NiO film III, respectively (Table S1, Supporting Information). The X‐ray diffraction (XRD) patterns of these NiO films are shown in **Figure** **1**a. The peaks at 37.3, 43.3, 63.0, 75.4, and 79.4 are attributed to (111), (200), (220), (311), and (222) diffraction of NiO with a face‐centered cubic structure, respectively. Moreover, the (111) line diffraction intensity is found to be higher than that of other diffractions, suggesting a preferred (111) orientation. Only diffraction line of (111) is observed in the XRD pattern of NiO film I, which reveal that the NiO film I has an unitary (111) plane orientation. According to the literature,^[^ ^50^ ^]^ texturing coefficient (111) (TC(111)) of NiO films II and III was calculated, the result is 1.38 and 1.27 respectively. Obviously, the texturing coefficient of (111) of NiO film is decreased with a reduction on the volume ratio of HOCH_2_‐CH_2_OH to H_2_O. Scanning electron microscopy (SEM) observations (Figure S1, Supporting Information) indicate that the three kinds of NiO films consist of large quantities of NiO rectangular nanoparticles or nanosheets, as shown in Figure S1a–c (Supporting Information). The NiO films I, II and III have essentially the same thickness, and the thickness is ≈130 nm (Figure S1d–f, Supporting Information). Figure 1b displays a transmission electron microscopy (TEM) image of NiO film I. Clearly, the NiO film I consist of a lot of NiO rectangular nanoparticles. The rectangular nanoparticles are actually assembled from NiO nanosheets. Figure 1c,d shows the high‐resolution TEM (HRTEM) images from box І and box II in (b) respectively. The lattice spacing of 0.15 nm corresponds to {220} planes of the face‐centered cubic structure NiO. Figure 1e,f shows the fast fourier transform patterns from HRTEM images in (c) and (d), respectively. The transform patterns are assigned to the cubic phase [111] zone axis of NiO. The TEM results show that the NiO film has a preferential [111] orientation. The bottom and top surfaces of NiO film are $\left(\bar{1}\bar{1}\bar{1}\right)$ and (111), respectively, and the crystal orientation is illustrated in Figure 1g.

### 2.2 (111) Orientation‐Dependent Piezoelectric Generation Behaviors

**Figure 2:**
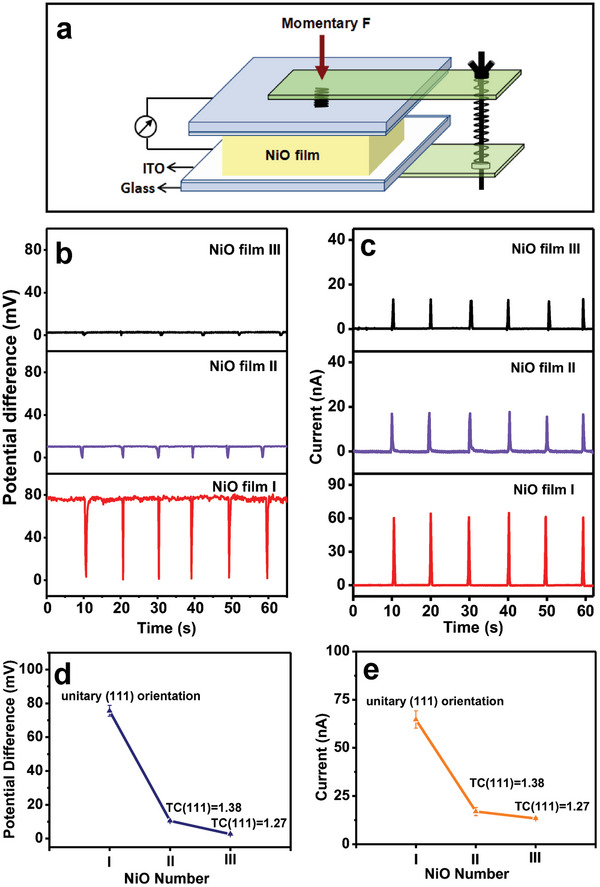
a) Schematic description of the NiO film piezoelectric generator. b–e) Measured electric potential difference (b and d) and output current (c and e) of the nanogenerators based on NiO films І, II, and III, which were cyclically compressed.

**Figure 3:**
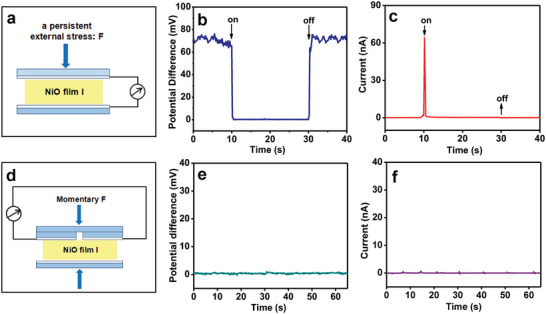
a) The measurement schematic diagram. b,c) Measured electric potential difference and output current of the NiO film І piezoelectric generator under a persistent compression. d) Schematic diagram of the current sensing measurement. e,f) Measured electric potential difference and output current of the NiO film І, which were cyclically compressed.

**Figure** **2**a displays a schematic description of piezoelectric generator based on NiO film. At a stress‐free status, the bottom and top surfaces of the NiO thin films have different potential, and the potential difference of NiO films І, II, and III was measured to be 75.6 ± 3.2, 10.5 ± 0.25, and 2.7 ± 0.46 mV, respectively (Figure 2b). When the NiO thin film is compressed, the potential difference decreases rapidly to zero and returns quickly to the original value with remove of the external stress. Figure 2c shows the measured output electric current. The output electric currents of the NiO film І, II, and III generators under periodic compression are 64.7 ± 4.5 nA, 17 ± 2.1 nA, and 13.4 ± 0.5 nA, respectively. It is evident that the potential difference and piezoelectric current both increase with an increase in the texturing coefficient of (111) of NiO film (Figure 2d,e), and the NiO film I with a unitary (111) orientation exhibits the highest piezoelectric generation performance. The piezoelectric generation behavior of NiO film I under a persistent compression was studied. The measurement schematic description is shown in **Figure** **3**a. When a persistent pressure is applied, the potential difference of 75.6 mV lowers rapidly to zero, and is kept at zero. The potential difference was recovered quickly to 75.6 mV with remove of the applied pressure, as shown in Figure 3b. When NiO thin film is compressed, a piezoelectric current of 64.7 nA is created. However, when the compression is maintained and removed, no electric current is seen (Figure 3c). Therefore, piezoelectric current is produced only at the moment when the NiO film is compressed. Furthermore, piezoelectric generation property on the top surface of NiO film I was investigated. The measurement schematic description is shown in Figure 3d. As both Indium Tin Oxide (ITO) electrodes were on the (111) surface of NiO film (Figure 3d), the potential difference is almost zero, and it is almost impervious to external stress (Figure 3e). The output piezoelectric current is very small (0.4 nA) (Figure 3f), much <64.7 nA. The results show that output currents can be observed only when the two ITO electrodes are on the (111) and $\left(\bar{1}\bar{1}\bar{1}\right)$ surfaces of NiO, respectively.

Moreover, the NiO films with different [111] orientations were prepared on a nickle substrate by the sol‐gel process. The XRD patterns of the as‐prepared NiO films are given in Figure S2 (Supporting Information). Clearly, the NiO film I on nickle substrate still has a unitary (111) plane orientation. The texturing coefficient of (111) of the NiO films II and III on the nickle substrate was estimated to be 1.37 and 1.25, respectively (Table S2, Supporting Information). The piezoelectric coefficient (d_33_) of direct piezoelectric effect of the NiO films I, II, and III are measured to be 13.1, 10.9, and 5.8 pC N^−1^, respectively. The piezoelectric coefficient of the NiO film I is higher than that of PbTiO_3_ film of 12 pC N^−1^ reported in the literature.^[^ ^51^ ^]^ In addition, inverse piezoelectric effect of the NiO films with different [111] orientations was also characterized by piezoresponse force microscopy(PFM). The atomic force microscopy(AFM) images of the NiO thin films are shown in Figure S3a–c (Supporting Information), which indicates the NiO films are assembled from particles or nanosheets. The results are consistent with the SEM observation in Figure S1 (Supporting Information). PFM phase images in Figure S3d–f (Supporting Information) indicate the spontaneous polarization orientation increasenes with an increase in the texturing coefficient of (111) of NiO film,^[^ ^52^ ^]^ which suggests that the spontaneous polarization is most likely in the [111] direction of NiO. The displacements as a function of applied voltage for the NiO films are given Figure S3g–i (Supporting Information). The inverse piezoelectric coefficient is determined from the slope of the displacement versus applied voltage plot.The inverse piezoelectric coefficient (d_33_) of the NiO films I, II and III is measured to be 14.3, 2.5, and 1.5 pm V^−1^, respectively. The piezoelectric coefficient of the NiO film I is larger than that of the 143 nm‐thick PbTiO_3_ films of 12.1 pm V^−1^^[^ ^53^ ^]^ and the bulk (0001) ZnO of 9.93 pm V^−1^.^[^ ^54^ ^]^ Like piezoelectric generation, the direct and inverse piezoelectric coefficients increase with an increase in the texturing coefficient of (111). These results show that the NiO films with [111] orientation are indeed piezoelectric.

### 2.3 (111) Orientation‐Dependent Photovoltaic and Rectifying Properties

**Figure 4:**
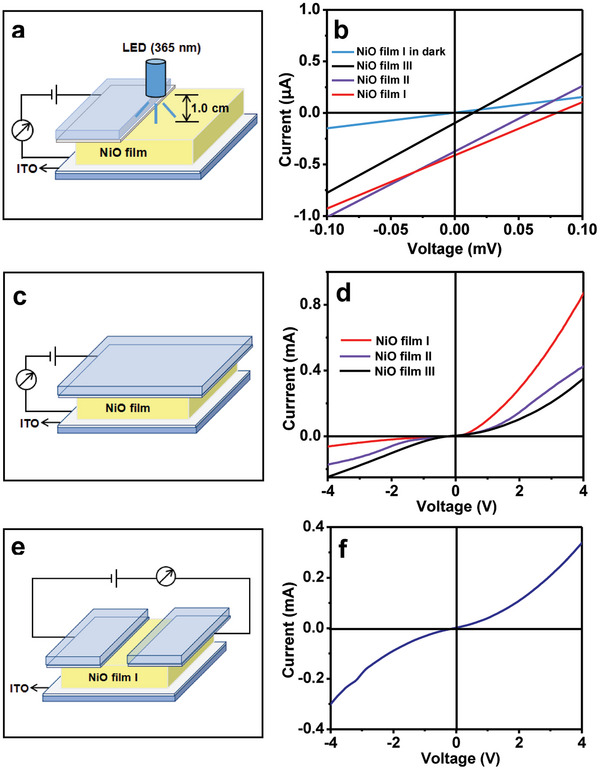
a) Schematic diagram of the NiO film photovoltaic device. b) Photovoltaic properties of the NiO films І, II, and III under illumination of an UV (365 nm) light emitting diode (LED) lamp (3.0 W). The light intensity is 0.158 W cm^−2^. c) The schematic diagram for electron transport property measurement of the NiO films. d) The measured current‐voltage curves of NiO film І, II, and III in the darkness. e) the current measurement schematic diagram. f) Dark current–voltage curve of the NiO film І measured from (e).

Absorption spectra of the NiO films I, II, and III are shown in Figure S4 (Supporting Information). It is obvious that the NiO thin films absorb significantly ultraviolet light at the wavelength of 300–400 nm. Therefore, a 365 nm ultraviolet light was employed as an excitation light to study the bulk photovoltaic characteristics of NiO films. The photovoltaic device schematic diagram, corresponding photograph, and measured current‐voltage curves are given in **Figure** **4**a,b and Figure S5 (Supporting Information), respectively. Clearly, the (111) orientated NiO films show the bulk photovoltaic property. The open‐circuit voltages (Voc) of NiO films I, II, and III are measured to be 0.079, 0.058, and 0.014 mV, respectively (Figure S6a, Supporting Information). Their short‐circuit current (Isc) is 0.413, 0.369, and 0.100 µA, respectively (Figure S6b, Supporting Information). Like piezoelectric property, the photovoltaic property of NiO thin films is enhanced by increasing (111) orientation.

In addition, the electron transport properties of the NiO films I, II, and III in the darkness were investigated. The current measurement schematic description and the measured results are given in Figure 4c,d, respectively. The NiO film I exhibits a typical rectifying characteristics of diode. That is, the current in forward bias is much larger than that in reverse bias. And the forward current is reduced and reverse current is increased by lowering (111) orientation, as shown in Figure 4d. Resistances of the NiO films were gauged under forward and reverse bias. For the NiO films I, II, and III, the forward resistance (R_f_) is 3.5, 7.2, and 8.3 KΩ (Figure S7a, Supporting Information), respectively, and the reverse resistance (R_r_) is 42.8, 32.9, and 13.4 KΩ (Figure S7b, Supporting Information), respectively. The ratio of R_r_ to R_f_ was found to be 12.2:1, 4.6:1, and 1.6:1 (Table S3, Supporting Information), respectively. When both ITO electrodes were on the (111) surface of NiO film I (Figure 4e), the measured current–voltage (*I*–*V*) curve is basically symmetric with respect to the origin. Namely, the *R*_f_ and *R*_r_ are almost the same, as shown in Figure 4f. The results show that the rectifying, photovoltaic, and piezoelectric properties of NiO films are closely related to the (111) orientation. The NiO film with a unitary (111) orientation exhibits the enhanced rectifying, photovoltaic, and piezoelectric generation properties.

### 2.4 Effect of Pressure on the Piezoelectric, Photovoltaic and Rectifying Properties

**Figure 5:**
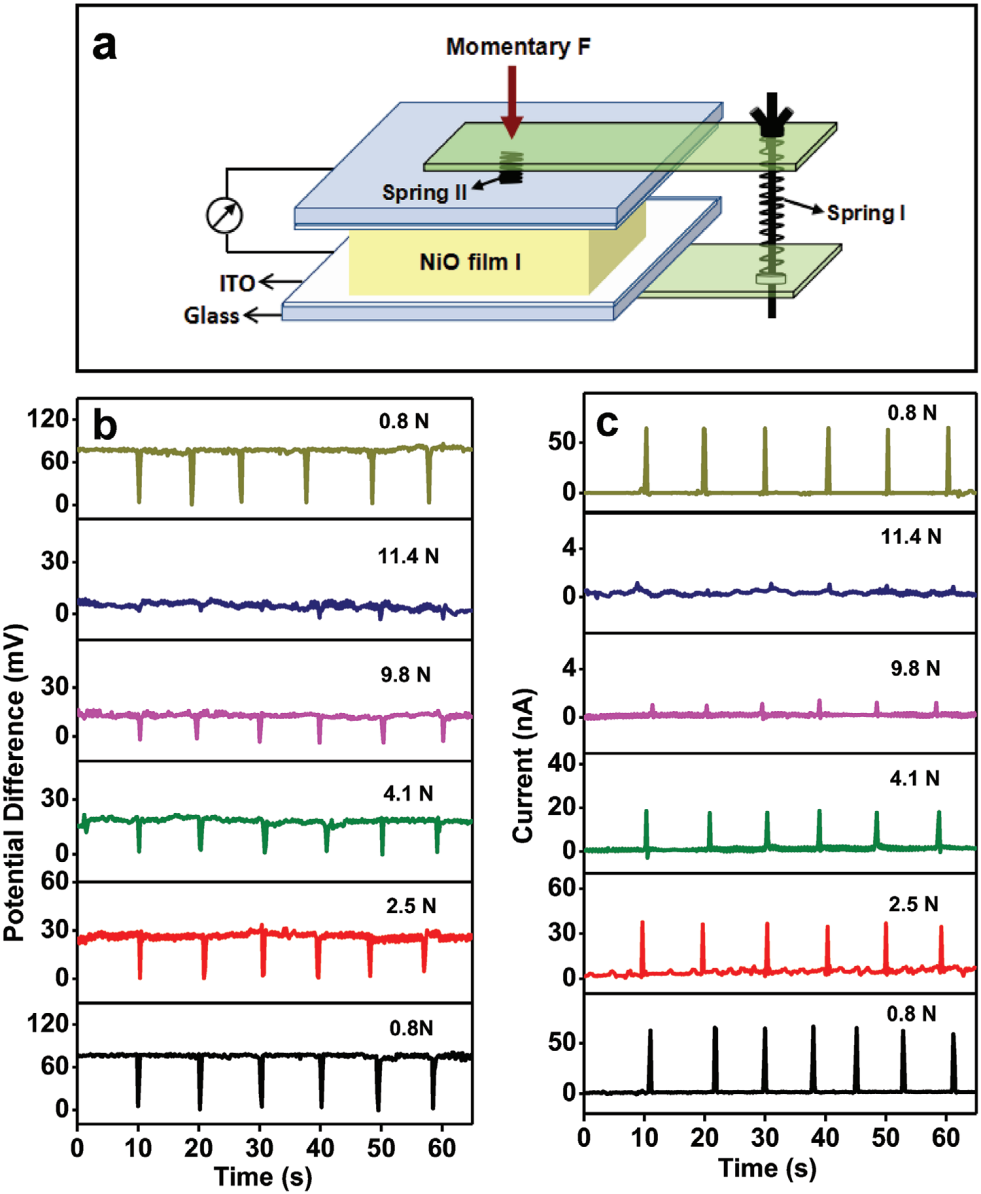
a) Schematic diagram of the NiO film piezoelectric generator in the presence of a persistent external stress. The persistent external stress is determined by measuring the deformation of spring II.The control over the external stress is achieved by adjusting spring I. The force bearing area is 3.0 cm^2^. b) Measured electric potential difference and c) output current of the NiO film I piezoelectric generator under the different persistent external forces.

In order to understand the piezoelectric mechanism, effects of pressure on the piezoelectric, photovoltaic and rectifying properties of NiO film I with the unitary (111) orientation were investigated. **Figure** **5**a–c and Figure S8 (Supporting Information) show the schematic description of piezoelectric property measurement under a sustained stress, corresponding photograph, and measured results, respectively. Clearly, when the sustained stress from 0.8 increased to 11.4 N, the potential difference between (111) and $\left(\bar{1}\bar{1}\bar{1}\right)$ planes of NiO film decreased from 76 ± 3.4 mV to 7.0 ± 2.2 mV. As the sustained stress increased from 0.8 to 11.4 N, the piezoelectric current is reduced from 64.7 ± 2.2 nA to 0.71 ± 0.42 nA. The presence of the sustained stress leads to a reduction in the output piezoelectric current. When the sustained external force decreased from 11.4 to 0.8 N, the potential difference was restored to the initial value(75.5 ±3.2 mV), the piezoelectric current increased to 64.0 ± 1.5 nA, as shown Figure S9a,b (Supporting Information). Clearly, the piezoelectric generation performance can be significantly reduced by applying a sustained stress, and this change is reversible.

**Figure 6:**
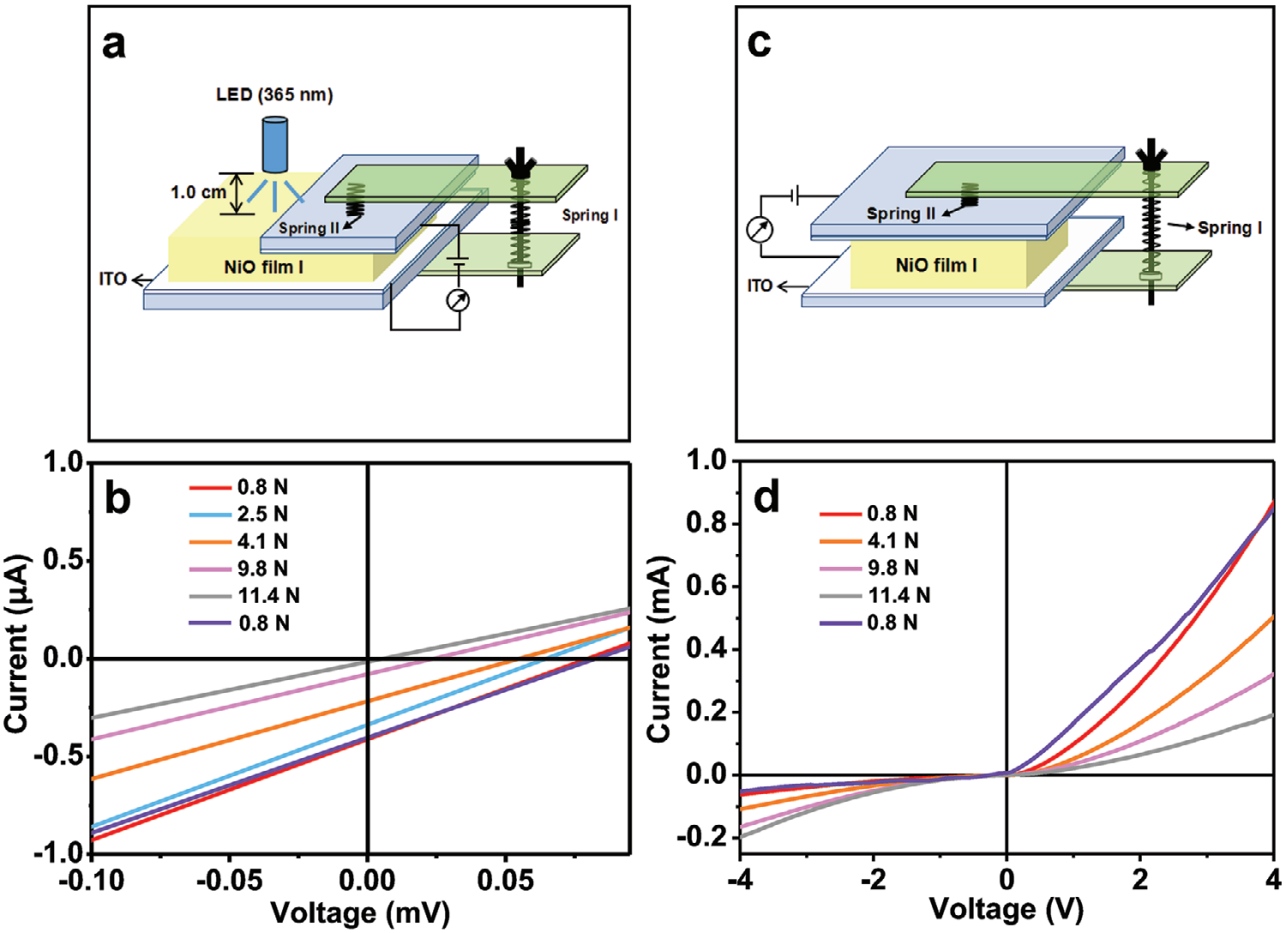
a) Schematic diagram of the NiO film photovoltaic device in the presence of a persistent external stress. b) Photovoltaic properties of the NiO film І under illumination of UV (365) nm light with the light intensity of 0.158 W cm^−2^ in the presence of a sustained external stress. The force bearing area is 2.0 cm^2^. c) Schematic diagram of current sensing measurements in the presence of a sustained external stress. The force bearing area is 3.0 cm^2^. d) Dark *I*–*V* curve of NiO film І under the different persistent external forces.

**Figure** **6**a,b shows the schematic description of photovoltaic property measurement under the sustained stress and measured result, respectively. Evidently, like the piezoelectric generation property, the presence of the sustained stress lowers obviously the photovoltaic property. When the applied stress augmented from 0.8 to 11.4 N, the Voc is reduced from 0.08 to 0.006 mV (Figure S10a, Supporting Information), the Isc is decreased from 0.414 to 0.02 µA (Figure S10b, Supporting Information). As the applied stress decreased from 11.4 to 0.8 N, the Voc and Isc were restored to their initial values (Figure 6b; Figure S10, Supporting Information). Figure 6c,d shows the schematic description of rectifying measurement under the sustained stress and measured result, respectively. It is found that the forward current decreased and the reverse current increased with increasing the sustained stress, Finally, a nearly symmetric *I*–*V* curve is observed (Figure 6d). When the applied stress augmented from 0.8 to 11.4 N, the forward resistance increased from 3.5 to 13.8 KΩ (Figure S11a, Supporting Information), the reverse resistance decreased from 46.6 to 15.9 KΩ (Figure S11b, Supporting Information). And the ratio of *R*_r_ to *R*_f_ was decreased from 13.4:1 to 1.1:1(Table S4, Supporting Information). When the applied stress is reduced from 11.4 to 0.8 N, R_r_ and R_f_ were restored to their original values. Like the piezoelectric generation and photovoltaic properties, the rectifying property can be regulated by applying a sustained stress, and this regulation is reversible. These results show that the rectifying, bulk photovoltaic, and piezoelectric behaviors of NiO film are not only related to its (111) orientation, but also to the applied stress. The application may cause a change in NiO crystal structure. The origins of the rectification, bulk photovoltaic, and piezoelectric effects may be the same.

### 2.5 The Atomic Mechanism of Photovoltaic and Rectification Effects

**Figure 7:**
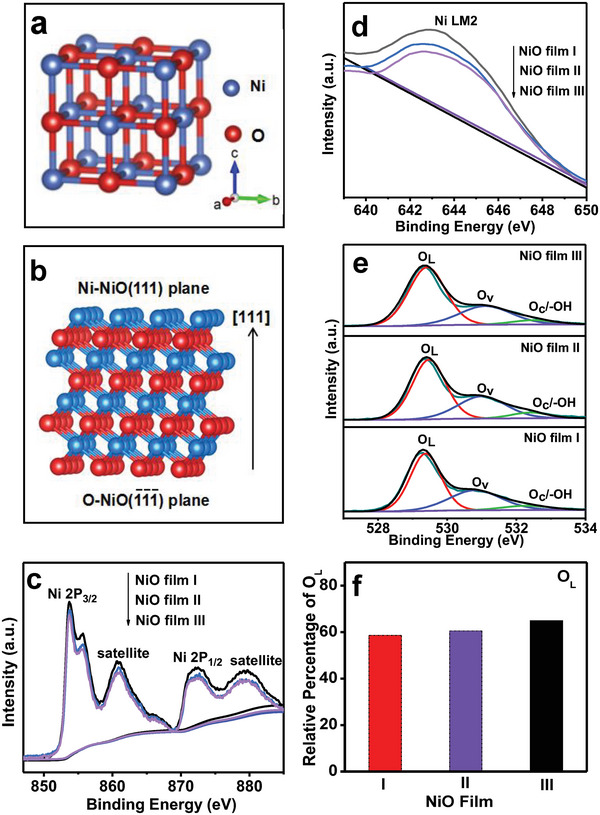
a) Crystal structure of the cubic phace NiO. b) Structure at atomic scale of NiO in the [111] direction. c) Ni 2p, d) Ni LM2, and e) O1s spectra of the NiO films І, II, and III. f) the relative percentage of O_L_.

According to the literature,^[^ ^55^ ^]^ the crystal structure and the atomic arrangement in the [111] direction of face‐centered cubic phase NiO are shown in **Figure** **7**a,b, respectively. Clearly, the [111] of NiO is a polar direction. The exposed NiO {111} polar crystal faces include a terminated (111) plane with Ni and a terminated $\left(\bar{1}\bar{1}\bar{1}\right)$ plane with O. To further verify this polar structure of NiO [111] direction, X‐ray photoelectron spectroscopy (XPS) spectra of the as‐prepared NiO films I, II, and III were measured. The results are given in Figure 7c–e and Figure S12 (Supporting Information). In the survey spectra of the three NiO films (Figure S12, Supporting Information), Ni 2p, Ni LM2, Ni 2s, Ni 3p3, and O1s peaks were observed, suggesting that the films are composed of Ni and O elements. Figure 7c,d displays the Ni 2p and Ni LM2 spectra of NiO films I, II, and III, respectively, which indicates that the amount of Ni on the surface changes in the order of NiO film I > NiO film II > NiO film III. Figure 7e shows O1s peaks of the three kinds of NiO films. Clearly, each O1s peak can be decomposed into three peaks. The peaks at 533.1, 531.4, and 529.3 eV are indexed to adsorbed oxygen (O_C_) / OH,^[^ ^56^ ^]^ vacancy oxygen (O_V_),^[^ ^57^ ^]^ and lattice oxygen (O_L_),^[^ ^58^ ^]^ respectively. The relative percentage of O_L_ of three NiO films was calculated. The results are given in Figure 7f. It is obvious that the relative percentage of O_L_ changes in the sequence of NiO film I < NiO film II < NiO film III. For NiO film I, NiO film II and NiO film III, the atomic ratio of Ni to O_L_ is estimated to be 1.34:1, 1.27:1, and 1.19:1, respectively (Table S5, Supporting Information). Obviously, the atomic ratio of Ni to O_L_ is >1:1, and increased with improving (111) orientation. The results provide experimental support for the polar structure of [111] direction of NiO shown in Figure 7b.

**Figure 8:**
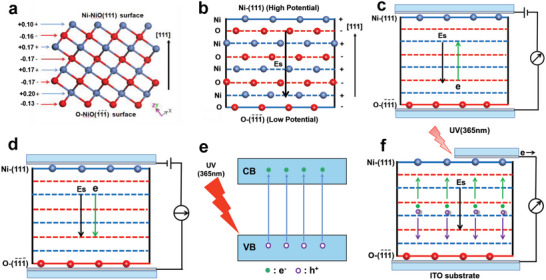
a) Atomic arrangement and atomic charge of NiO in the [111] direction. b) Schematic diagram of Es in NiO thin film with a unitary (111) orientation. Schematic diagram of c) forward‐biased and d) reverse‐biased [111] orientated NiO thin films. e) Generation of the photogenerated electrons and holes. f) Flow of the photogenerated charges driven by the Es.

According to the literature,^[^ ^50^ ^]^ the atomic arrangement of the [111] direction of face‐centered cubic phase NiO are shown in **Figure** **8**a. Obviously, in the [111] polar direction, the Ni and O ions are not located on the same layer, and the Ni layer and O layer are arranged alternately. In order to determine this polar structure of NiO in the [111] direction, the NiO octahedrons with exposed {111} facets are prepared by the method reported in our previous literature.^[^ ^50^ ^]^ The atomic scale structure along the [111] direction of NiO octahedron was investigated using aberration‐corrected scanning transmission electron microscopy (STEM). The aberration‐corrected TEM images at different magnification are given in Figure S13 (Supporting Information). Clearly, in the [111] directionof NiO, the Ni layer and O layer are exactly arranged alternately, which is the same as structure in Figure 8a obtained by density functional theory calculations. The Ni and O ions are positively and negatively charged, respectively. The charge center of positive Ni is above that of negative O in the [111] polar direction. That is, polarization has occurred. As a result, a dipole moment is produced and an electric field (Es) is spontaneously formed between the negatively charged O‐terminated (O‐$\left(\bar{1}\bar{1}\bar{1}\right)$) surface and positively charged Ni‐terminated (111) (Ni‐(111)) surface. The direction of E_S_ is from Ni‐(111) to the O‐$\left(\bar{1}\bar{1}\bar{1}\right)$ surface, as shown in Figure 8b. The Ni‐(111) surface has higher potential than O‐$\left(\bar{1}\bar{1}\bar{1}\right)$, and thus, there is a potential difference between the Ni‐(111) and O‐$\left(\bar{1}\bar{1}\bar{1}\right)$ surfaces, and this potential difference is called the spontaneous voltage (Vs). Therefore, at a stress‐free status, the potential difference (voltage) between the NiO film top and bottom surfaces increases with enhancing [111] orientation. The existence of Es/Vs is the physical basis of bulk photovoltaic, rectifying, and piezoelectric effects. For the rectifying effect, when the applied bias is in the same direction as the E_S_/Vs, the Es/Vs promotes electron migration. A smaller resistance and a larger current are thus observed under forward bias (Figure 8c). In reverse bias, Es and the bias voltage are in the opposite direction. It inhibits electron migration, a larger resistance, and a smaller current thus are observed (Figure 8d). For the bulk photovoltaic effect, when (111)‐oriented NiO film is irradiated by 365 nm UV light, electrons are excited from the valence band (VB) to the conduction band (CB) (Figure 8e). Thus electron (e^−^) and hole (h^+^) are produced in the NiO film. Under the action of Es/Vs, the photoproduced e^−^ and h^+^ move toward Ni‐(111) and O‐$\left(\bar{1}\bar{1}\bar{1}\right)$ plane, respectively. When the (111) and $\left(\bar{1}\bar{1}\bar{1}\right)$ surfaces of the NiO film are connected to the external circuit, these photoproduced charges are collected, and the bulk photovoltaic effect is observed (Figure 8f).

### 2.6 The Pressure‐Induced Change in the Polar Structure of NiO Film

**Figure 9:**
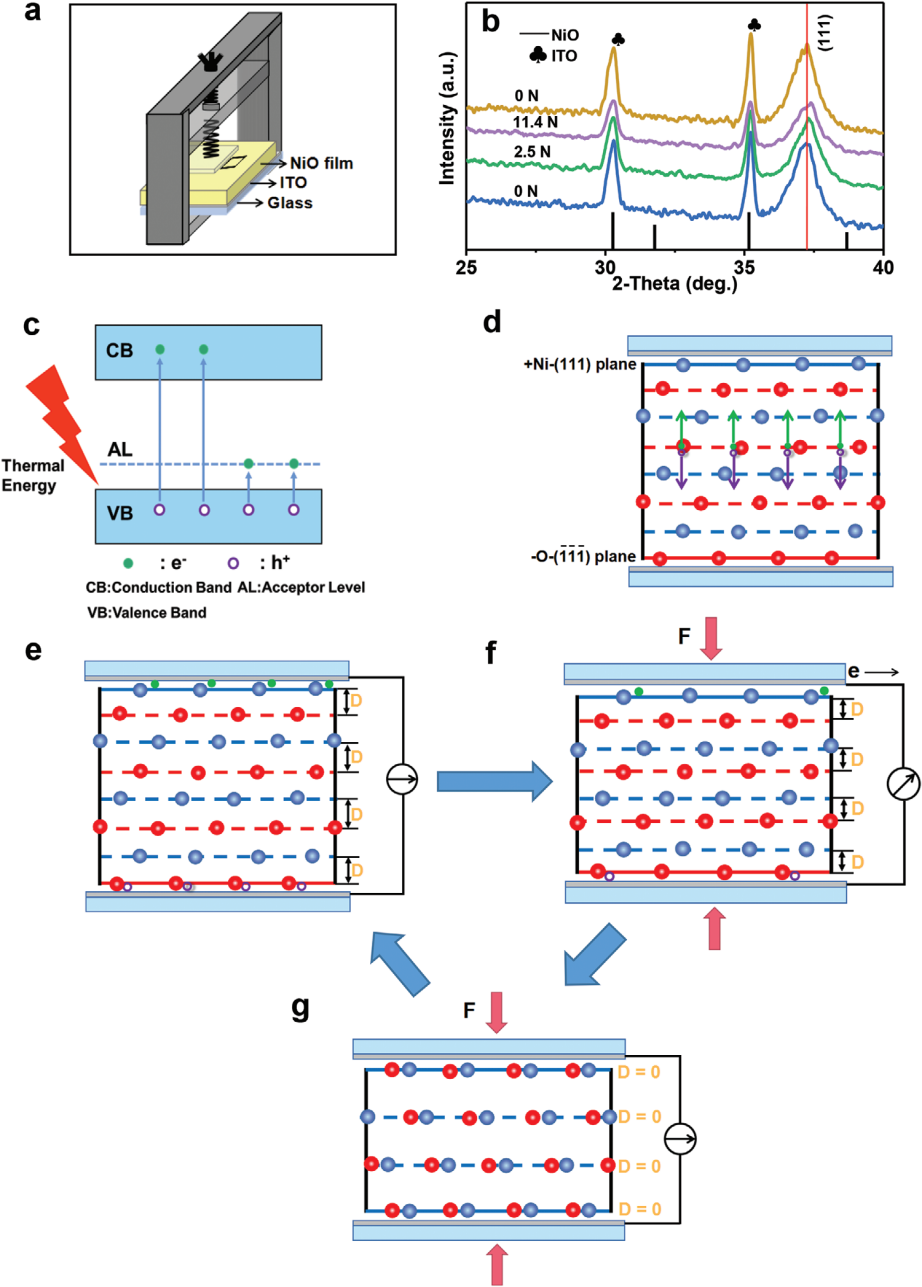
a) Schematic diagram of the strain sample under a persistent external stress for XRD measurement. b) XRD patterns measured from box in (a) under different external stresses. c) Formation of the thermal generated electrons and holes. d) Flow of the thermal generated charges driven by the E_S_. e–g) Schematic illustration of physical mechanism at atomic level of the NiO thin film nanogenerator.

To understand the piezoelectric effect and the effect of pressure on the piezoelectric, rectifying, and photovoltaic properties, the sustained stress influence on the crystal structure of NiO film I with unitary (111) orientation was investigated. The measurement schematic description under a sustained stress and corresponding photograph are shown in **Figure** **9**a and Figure S14 (Supporting Information), respectively. As shown in Figure 9a, when a persistent external stress is applied on the NiO film, the XRD patterns of the strain NiO film were measured. The as‐obtained results are given in Figure 9b. It is clear that when the applied stress increased from 0 to 11.4 N, line of the NiO (111) diffraction moved from 37.25 to 37.39. The interplanar spacing of (111) plane from 2.412 decreased to 2.404, indicating that the applied external stress can reduce the spacing between the (111) crystal faces. When the applied stress was removed, the line of the NiO (111) returned to its original position, indicating this change in crystal structure is also reversible.

### 2.7 A Novel Atomic Mechanism of Piezoelectric Generation

On the basis of the above experimental results, we present a novel piezoelectric generation atomic mechanism. Figure 9c shows the schematic diagram for energy level structure of NiO p‐type semiconductor. Some electrons in the VB are excited to the CB and acceptor level owing to thermal excitation at room temperature, some electrons and holes are thus generated in NiO films. Under the action of Es/Vs, these electrons and holes move to the Ni‐(111) and O‐$\left(\bar{1}\bar{1}\bar{1}\right)$ face, respectively, as shown in Figure 9d, and are bound to the (111) and the $\left(\bar{1}\bar{1}\bar{1}\right)$ surfaces due to electrostatic action, respectively (Figure 9e). When the NiO films with high [111] orientation are compressed by applying a stress in [111] the direction, the structure evolution in the [111] direction is shown in Figure 9e–g. As shown in Figure 9e, each Ni ion is between the two O ions in the lower layer in the [111] direction. The distance (D) between Ni layer and O layer is reduced under action of the applied stress (Figure 9f). When the applied stress is >11.4 N, the D is almost zero. The Ni and O ions merge into the same layer (Figure 9g). And thus, the polar structure becomes the nonpolar structure. As a result, the spontaneous polarization disappears, and the Es and Vs become completely zero. When the strain NiO film is connected to an external circuit, the electron and holes trapped at the (111) and the $\left(\bar{1}\bar{1}\bar{1}\right)$ surfaces are released, and a transient current is observed. As the applied stress is released, the nonpolar structure (Figure 9g) reverts to the original polar structure (Figure 9e) under the action of lattice elastic force. And thus, the spontaneous polarization and dipole moment produced again, and the Es and Vs revert to their original values. The electron and holes trapped at the (111) and the $\left(\bar{1}\bar{1}\bar{1}\right)$ surfaces are produced again. When the NiO film with (111) orientation is periodically compressed, the NiO structure and Es/Vs change periodically, and thus a periodic piezoelectric current is generated. The trapped charges exist only on the polar surfaces of NiO. The output current can be thus observed only when the NiO film with the polar structure is compressed. When the applied stress is removed, the nonpolar structure of NiO changes into the polar structure, the trapped charges are created, and no output current is observed.

It is clear that the fundamental of the NiO film piezoelectric generator, bulk photovoltaic device, and rectifying diode relies on the presence of spontaneous polarization in the [111] direction of NiO. The Es in the NiO film with (111) orientation increases with increasing (111) orientation. And thus, NiO films with unitary (111) orientation show enhanced piezoelectric generation, bulk photovoltaic, and rectifying properties. When a sustained stress is applied on the NiO film with (111) orientation, the spontaneous polarization and Es are lowered, and thus the piezoelectric generation, bulk photovoltaic, and rectifying properties are decreased. When the applied stress is >11.4 N, the spontaneous polarization is vanished, as a result, the Es and the rectifying, piezoelectric, and bulk photovoltaic effects are completely faded away. The potential is the same at all positions on the Ni‐(111) surface. As both ITO electrodes are on the (111) surface of NiO film, the potential difference between two ITO electrodes is zero, and thus no piezoelectric, bulk photovoltaic, and rectifying effects were seen.

### 2.8 The Establishment of Polar Structure Piezoelectric Mechanism

Based on the above analysis and discussion, as for the piezoelectric effect, we have gained the following new understanding: 1) The piezoelectric effect originates from the spontaneous polarization/polar structure in materials rather than the non‐centrosymmetric crystal structure. This polarization can spontaneously produce an electric field or voltage, but can not produce an electric current. 2) In the direct piezoelectric effect, the applied stress can not polarize the material, but it can change the polar structure of the material. When the applied stress transforms the polar structure of the material into a non‐polar structure, a transient current is generated. Thus, in the direct piezoelectric effect, an electric current, rather than polarization or voltage, is produced. Therefore, the existing piezoelectric theory may be wrong. The piezoelectric potential that is considered to be the physical basis of the piezoelectric generators and piezotronics does not exist. 3) The piezoelectric generators, similar to capacitors, compression produces discharge, releasing pressure for charging. 4) the piezoelectric, bulk photovoltaic, and rectifying effects are always accompanied, and the three effects originate from the polar structure with E_S_. This will lead to the formation of a unified theory of polar structure with a wide range of implications.

We have observed the structure of the reported semiconductor piezoelectric nanogenerators in detail and found that the electrodes are always connected to (0001) Zn‐ZnO and O‐ZnO $\left(000\bar{1}\right)$ polar surfaces or [0001] polar direction in all ZnO piezoelectric nanogenerators reported.^[^ ^11^, ^12^, ^13^, ^14^, ^15^, ^16^, ^17^, ^18^, ^19^, ^20^, ^21^, ^22^, ^23^, ^24^, ^25^, ^26^, ^27^, ^28^, ^41^ ^]^ The electrodes are found to be linked to the Te‐(111) and Cd‐(111) surfaces in the CdTe nano/microwire nanogenerator.^[^ ^31^ ^]^ For the MoS_2_ monolayer piezoelectric device, only while the two electrodes are connected to S and Mo of MoS_2_ monolayer_,_ respectively, the piezoelectric device shows high piezoelectric response.^[^ ^59^ ^]^ The (111) polar orientation enhanced piezoelectric generation property is observed in CdO film with different (111) orientations.^[^ ^60^ ^]^ In addition, the polar‐orientated Co_3_O_4_ and PbTiO_3_ films have been found to demonstrate enhanced piezoelectric generation and bulk photovoltaic properties and these studies are being continued. The results fully illustrate that the piezoelectric effect originates from the polar structure rather than the non‐centrosymmetric structure. The piezoelectric mechanism originating polar structure may be the only correct one.

## 3 Conclusion

Centrosymmetric NiO crystal is generally considered to have no piezoelectric properties. We demonstrate a (111)‐oriented NiO film piezoelectric generator. The piezoelectric generation, rectifying, and photovoltaic properties of NiO films are improved by increasing polar (111) orientation, and attenuated by applying a sustained stress on the NiO film. The presence of Es in polar (111) oriented NiO film is found to be the physical basis of the piezoelectric generators, rectifying effect, and bulk photovoltaic effect. Thus, we describe NiO piezoelectric, rectifying, and bulk photovoltaic mechanisms at the atomic level. The mechanism may rewrite the current piezoelectric theory and will establish a unified theory of polar structure with wide implications. The polar‐orientated films can be used to fabricate piezoelectric generators and other optoelectronic devices with high performances.

## 4 Experimental Section

### Preparation of NiO Films

NiO films were prepared by spin‐coating NiO precursor sol and subsequent heat treatment. The flow chart of preparation is given in Figure S15 (Supporting Information). Preparation of NiO precursor sol: Nickel chloride hexahydrate (0.099 g) and ethylene glycol‐water mixed solvent (14.0 mL) were added to a beaker. The mixture was magnetically stirred for 10 min, ammonia (0.6 mL) was added and continuously stirred for 10 min, forming a clear solution. The solution was put a 50 mL stainless‐steel autoclave. The autoclave was heated at 130 °C for 24 h, and then cooled to room temperature, A light green gel was collected by centrifugation (10000 revolutions per minute (rpm)). The gel and ethanol (2.0 mL) were mixed, then irradiated by ultrasound for 30 min, forming a light green NiO precursor sol solution. An ITO coated glass slide (2.0 × 2.0 cm^2^) was washed in acetone, isopropyl alcohol, and absolute ethanol ultrasonic both for 15 min, respectively. 220 µL of NiO precursor sol solution was put on the ITO conductive glass. The ITO glass was rotated at 1500 rpm for 3 s, and at 4000 rpm for 20 s, then preheated at 300 °C for 10 min in air. This process was repeated five times. The as‐obtained film was heated in air at 500 °C for 2 h.

### Measurement of Piezoelectric, Rectifying, and Photovoltaic Properties

The NiO film with [111] orientation was clamped in two ITO glasses to form a NiO generator. The effective area of NiO thin films is ≈3.0 cm^2^ (2.0 × 1.5 cm), and corresponding photograph is displayed in Figure S16 (Supporting Information). The NiO film generator is driven by cyclic compression in [111] direction. The electrical property measurement was carried out on a digital source meter (2601, Keithley, USA) at room temperature. The photovoltaic performance was measured at room temperature under illumination of an ultraviolet light‐emitting diode lamp (365 nm, 3 W).

### Characterizations

The morphology and thickness of NiO films were examined by SEM on a scanning electron microscope (SU‐8020, Hitachi, Japan). The crystal orientation and polar structure of NiO films were examined by TEM, XRD, and XPS. The TEM measurement was carried out on a field emission transmission electron microscope (Tecnai G2 F20, FEI, America). The XRD spectra were recorded on a Smart Lab 9 X‐ray diffractometer using Cu Kα1 radiation (Rigaku, Japan). The XPS spectra were recorded using an XPS spectrometer (ESCALAB Xi+, Thermo Fischer Scientific). The binding energy was calibrated by using the binding energy of C1s(284.8 eV). Absorption spectra were recorded on a spectrophotometer (UV3600, Shimadzu, Japan). The direct piezoelectric coefficient of NiO films was measured by YE2730A d33 Meter (Sinocera). The inverse piezoelectric characterization of the NiO films was performed on a piezoelectric force microscope (Dimension ICON, Bruker, USA). The dark field‐STEM and ABF‐STEM observations were carried out on a double spherical aberration‐corrected JEOL JEM‐ARM200F microscope.

## Conflict of Interest

The authors declare no conflict of interest.

## Supporting information

Supporting Information

## Data Availability

The data that support the findings of this study are available from the corresponding author upon reasonable request.
