# Supplementary material for: Origin and Mechanism of Piezoelectric and Photovoltaic Effects in (111) Polar Orientated NiO Films
Source: Adv Sci (Weinh). 2023 Sep 26;10(32):2304637. doi: 10.1002/advs.202304637 (PMC10646231; doi:10.1002/advs.202304637)
Supplement: Supplementary file 1 — Supporting Information [file ADVS-10-2304637-s001.pdf]

ADVANCED SCIENCE
Open Access

Supporting Information

for *Adv. Sci.*, DOI 10.1002/advs.202304637

Origin and Mechanism of Piezoelectric and Photovoltaic Effects in (111) Polar Orientated NiO Films

Nana Fan, Yingfei Wang, Bin Liu, Heqing Yang* and Shengzhong Liu*

Supporting Information

Origin and mechanism of piezoelectric and photovoltaic effects in (111) polar orientated NiO films

Nana Fan, Yingfei Wang, Bin Liu, Heqing Yang* and Shengzhong Liu*

Supporting Information

Origin and mechanism of piezoelectric and photovoltaic effects in (111) polar orientated NiO films

Nana Fan[a,b], Yingfei Wang[a,b], Bin Liu[a,b], Heqing Yang[a,b]* and Shengzhong Liu[b,c]*

[a]Shaanxi Key Laboratory for Advanced Energy Devices, Shaanxi Engineering Laboratory for Advanced Energy Technology, Key Laboratory of Macromolecular Science of Shaanxi Province, School of Materials Science and Engineering,Xi'an, 710119, China

[b]Shaanxi Normal University

[c]Key Laboratory of Applied Surface and Colloid Chemistry, National Ministry of Education, Shaanxi Key Laboratory for Advanced Energy Devices, Shaanxi Engineering Lab for Advanced Energy Technology, School of Materials Science and Engineering,Xi'an 710119, China

This PDF file includes:

Figure S1-16 and Table S1-5

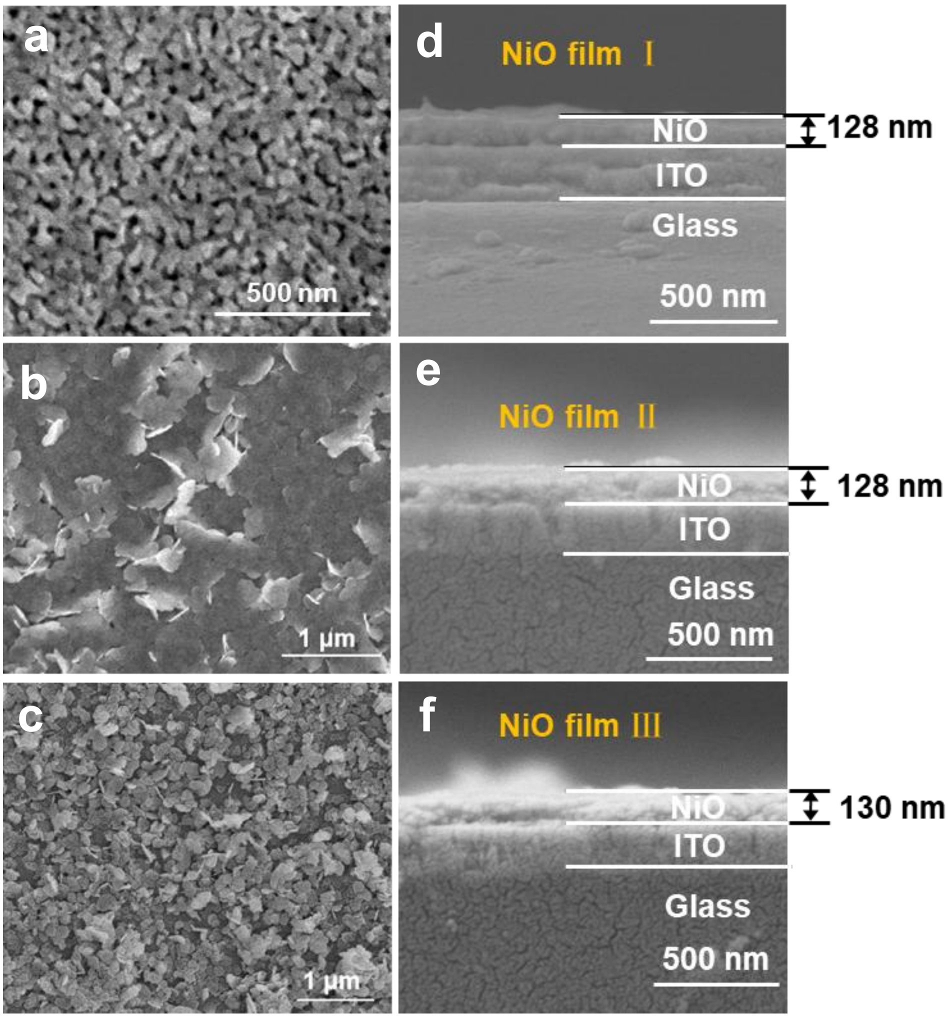

Figure S1. a-c) Top-view SEM and d-f) cross-sectional SEM images of the as-obtained NiO films I, II and III, respectively.

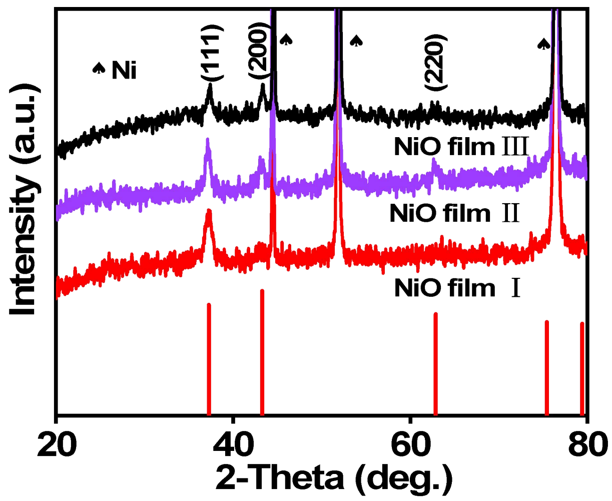

Figure S2. XRD patterns of the NiO films I, II and III on nickle substrate. The stick pattern is the standard XRD pattern of NiO powders with Cu $K_{\alpha 1}$ radiation (Joint Committee on Powder Diffraction Standards card file no. 47-1049).

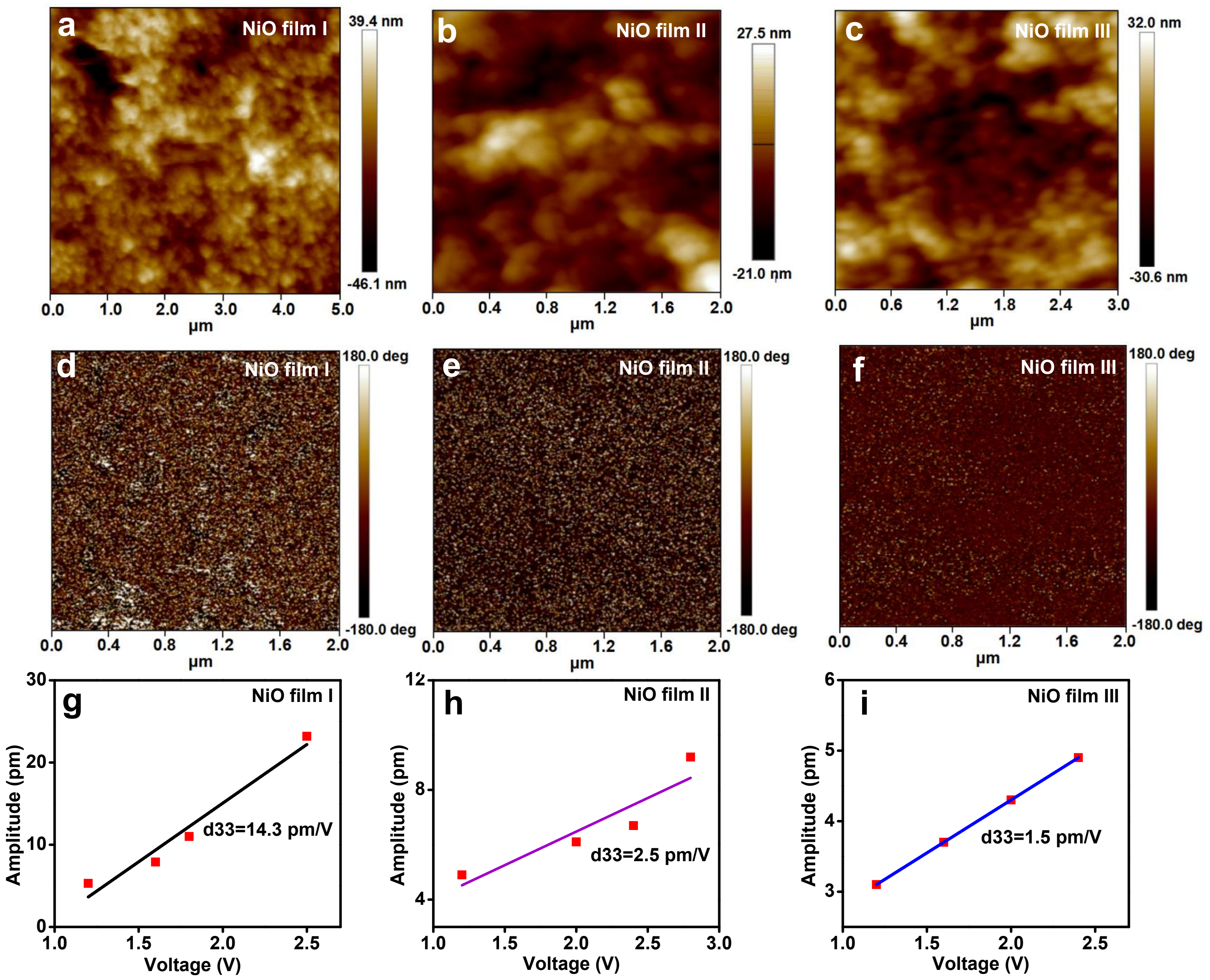

Figure S3. a-c) AFM images of the NiO films I, II and III on nickle substrate. d-f) PFM phase images of the NiO films I, II and III on nickle substrate. g-i) linear fitting of the displacement vs. applied AC voltage for the NiO films I, II and III on nickle substrate.

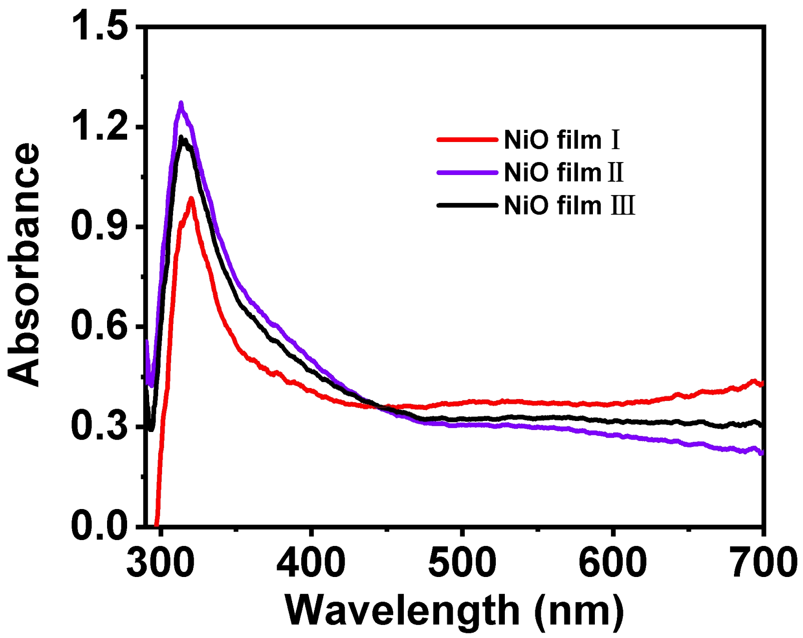

Figure S4. UV-vis absorption spectra of the NiO films I, II and III.

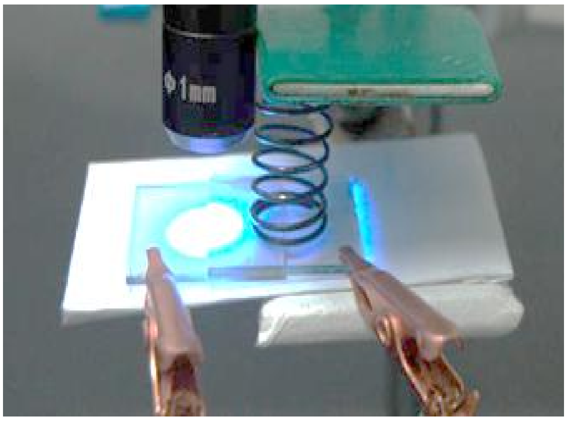

Figure S5. Photograph of the NiO film photovoltaic device.

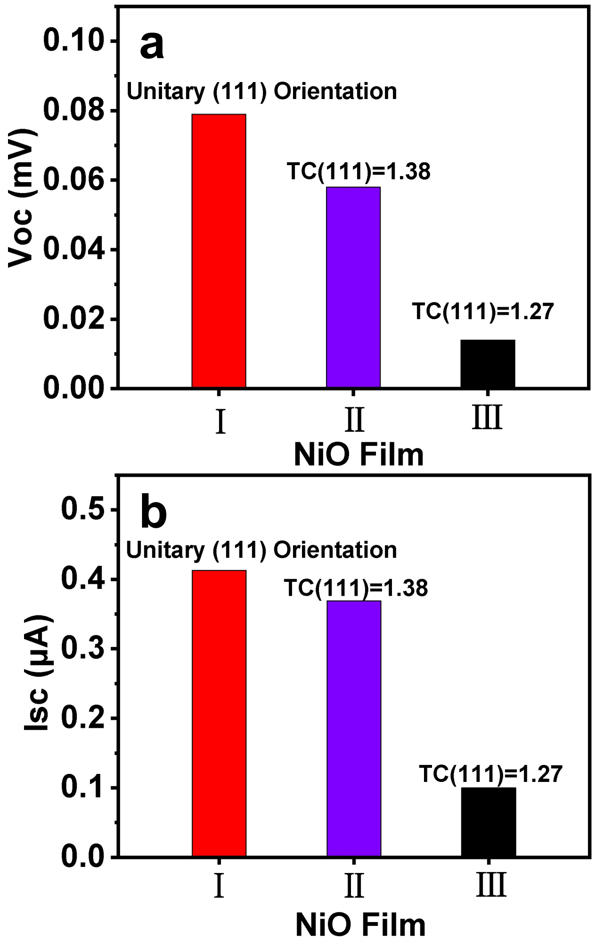

Figure S6. a,b) Voc and Isc of the NiO films I, II and III obtained from Figure 4a, respectively

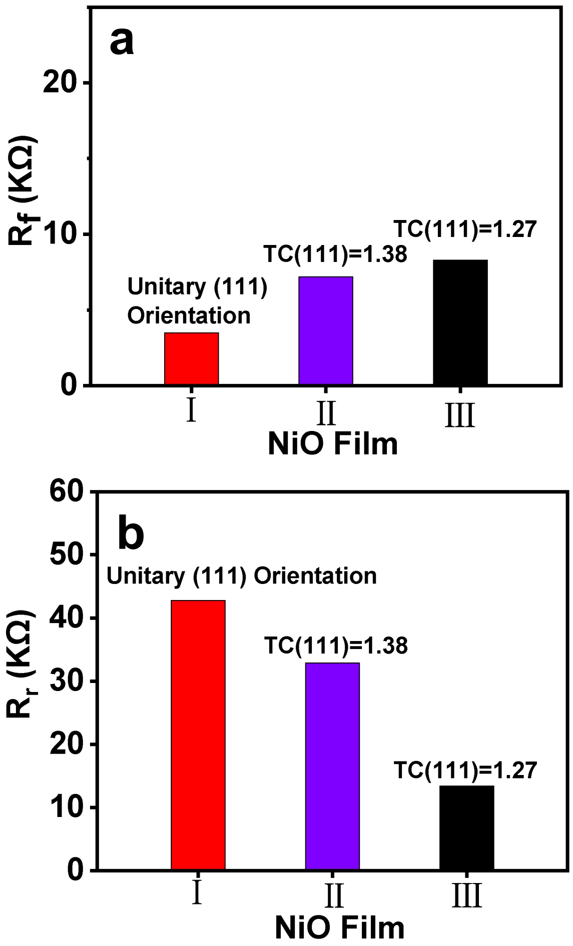

Figure S7. a,b) R_f and R_r of the NiO films I, II and III, obtained from Figure 4d. respectively.

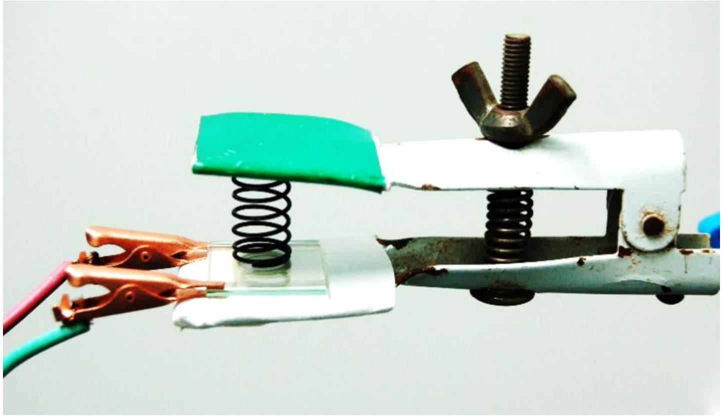

Figure S8. Photograph of the NiO film piezoelectric generator in the presence of a persistent external stress.

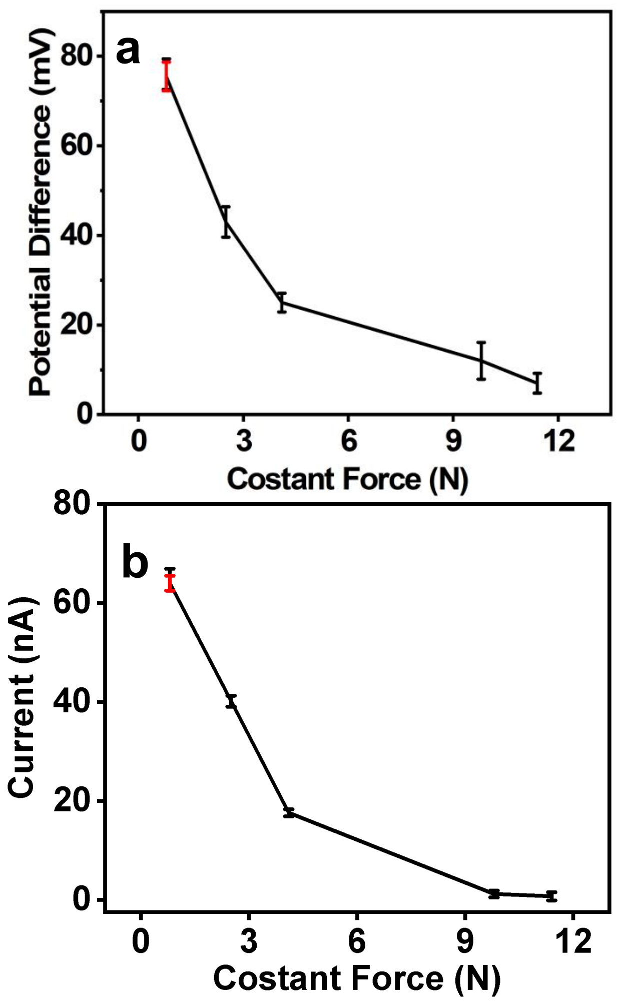

Figure S9. a) The plot of electric potential difference of the NiO film I piezoelectric generator versus the external force. b) The plot of output current of the NiO film I piezoelectric generator versus the external force. The force bearing area is 3.0 cm^2.

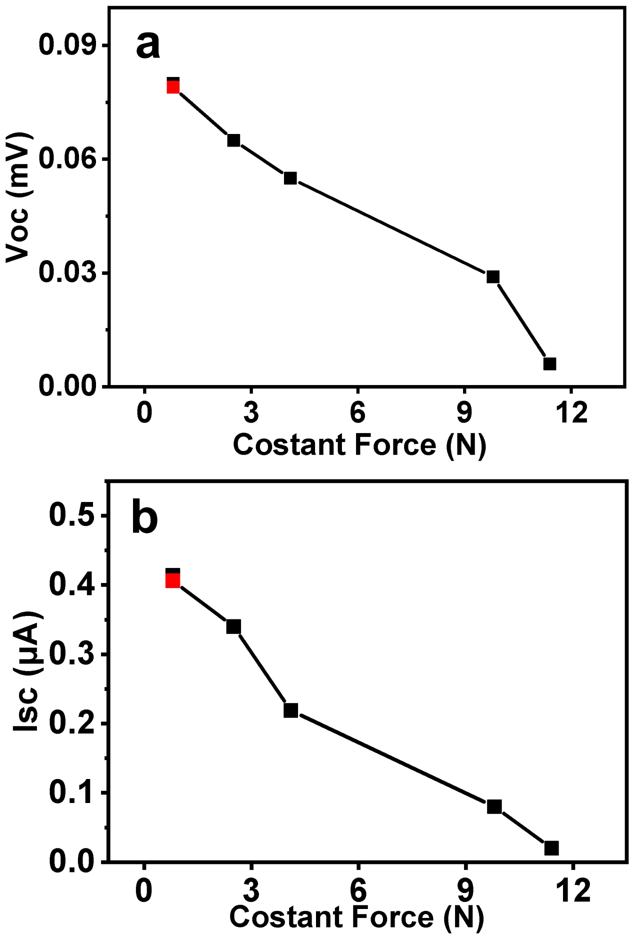

Figure S10. a) The plot of Voc of the NiO film I photovoltaic device versus the external force. b) The plot of Isc of the NiO film I photovoltaic device versus the external force. The force bearing area is 2.0 cm^2.

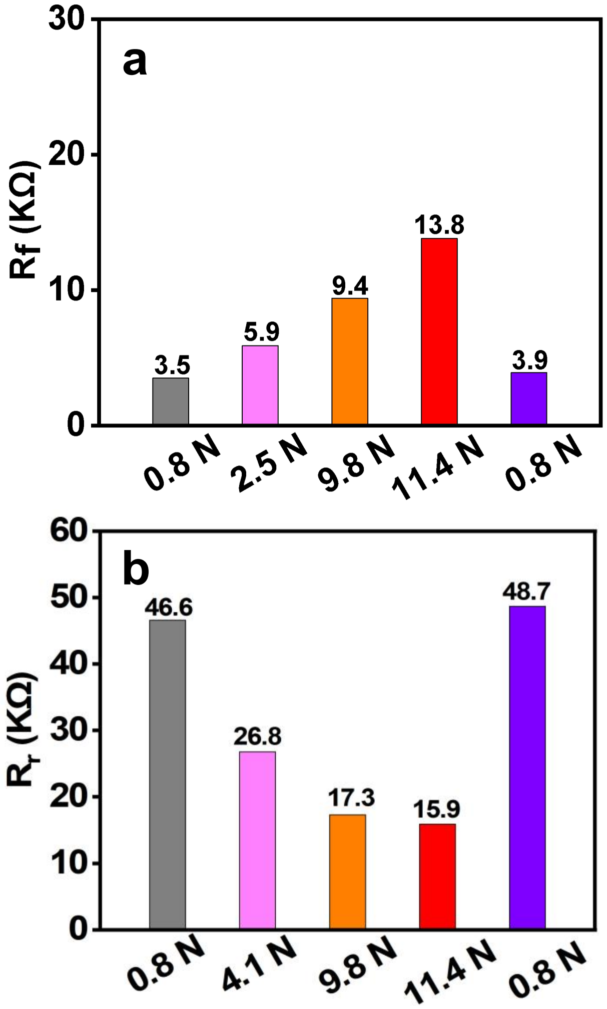

Figure S11. a) R_f and b) R_r of the NiO film I under different external forces obtained from Figure 6d. The force bearing area is 3.0 cm^2.

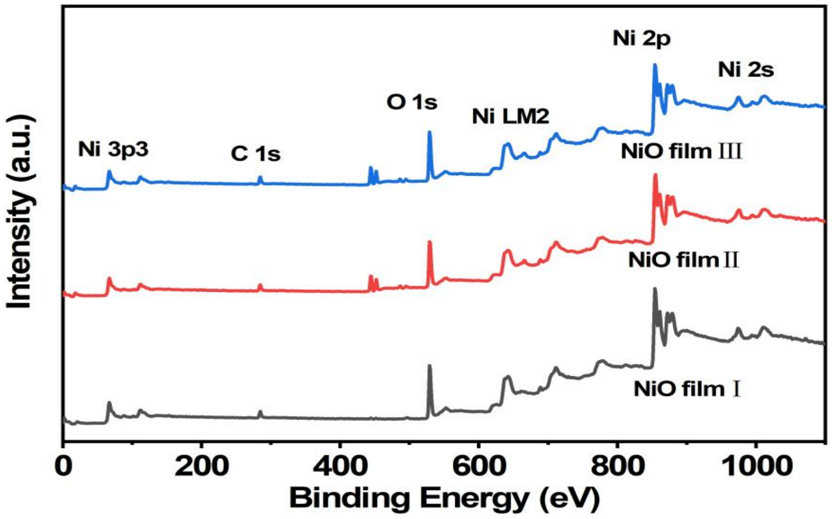

Figure S12. Survey XPS spectra of the NiO thin films I, II and III

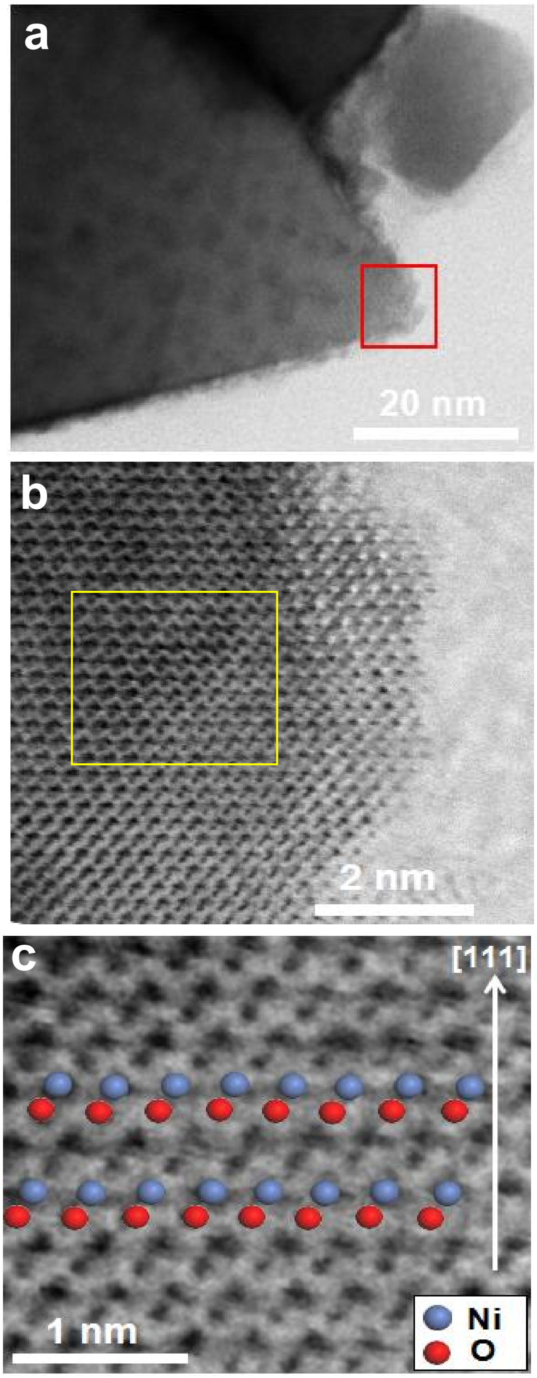

Figure S13. a) Annular bright field (ABF)-STEM general view of the studied NiO octahedrons. b) Atomic resolution aberration corrected ABF-STEM imag from the region marked with a yellow square in a). c) ABF-STEM magnified detail of the region marked with a yellow square in b).

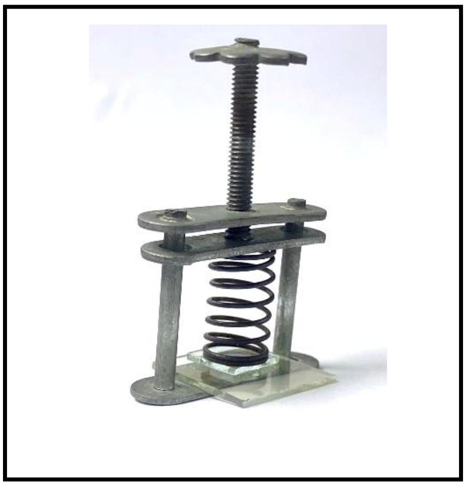

Figure S14. Photograph of the strain sample under a persistent external stress for XRD measurement. The force bearing area is 1 cm^2.

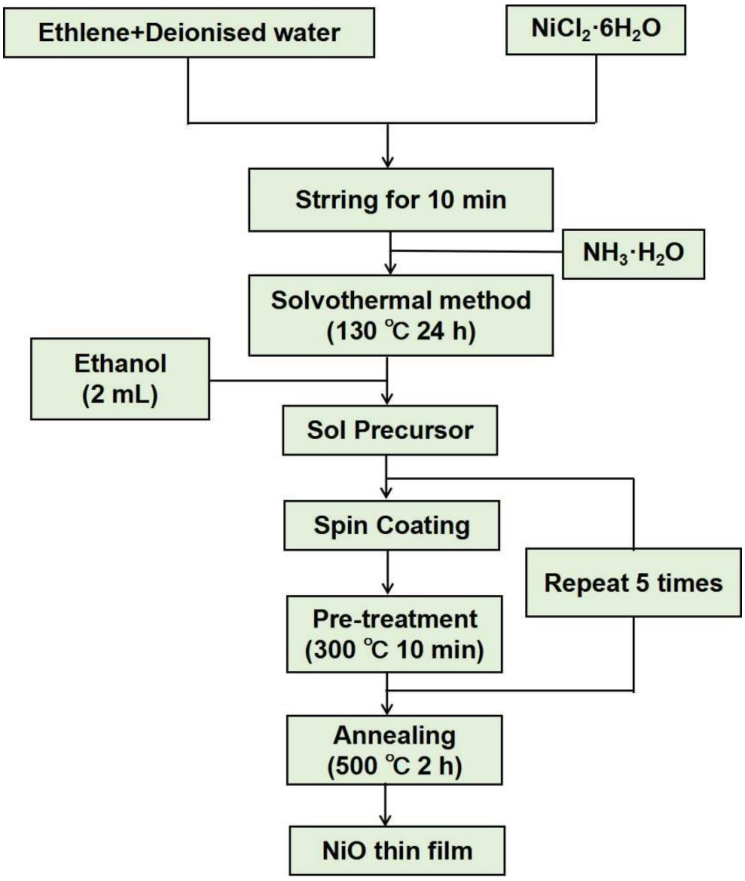

Figure S15. Synthetic process schematic diagram of NiO films.

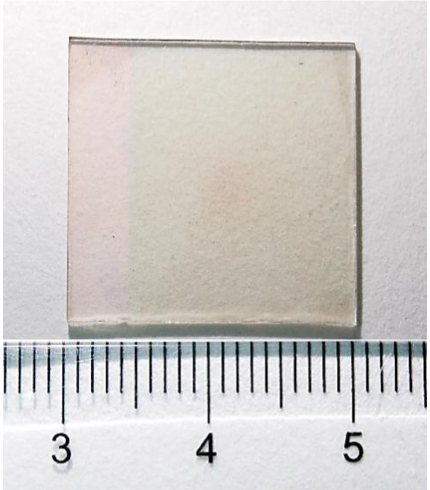

Figure S16. Photograph of the as-prepared NiO thin film.

Supplementary Tables

Table S1. The NiO films with different [111] orientations prepared on ITO glass substrate by adjusting the volume ratio of $HOCH_2$-CH_2OH to H_2O.

	NiO film I	NiO film II	NiO film III
Volume ratio of $HOCH_2$-CH_2OH to H_2O	2 : 1	1 : 13	0 : 14
TC(111)	Unitary (111) Orientation	1.38	1.27

Table S2. The NiO films with different [111] orientations prepared on nickle substrate by adjusting the volume ratio of $HOCH_2$-CH_2OH to H_2O.

	NiO film I	NiO film II	NiO film III
Volume ratio of $HOCH_2$-CH_2OH to H_2O	2 : 1	1 : 13	0 : 14
TC(111)	Unitary (111) Orientation	1.37	1.25

Table S3. The ratios of Rr to Rf of the NiO thin films I, II and III measured from Figure 4d.

	NiO film I	NiO film II	NiO film III
TC(111)	Unitary (111) Orientation	1.38	1.27
Ratio of R_r to R_f	12.2 : 1	4.6 : 1	1.6 : 1

Table S4. The ratios of R_r to R_f of the NiO film I under different external forces obtained from Figure 6d

External force (N)	0.8	4.1	9.8	11.4	0.8
Ratio of R_r to R_f	13.4 : 1	4.5 : 1	1.8 : 1	1.1 : 1	12.5 : 1

Table S5. The molar ratios of Ni to O_L of the NiO films I, II and III

	NiO film I	NiO film II	NiO film III
TC(111)	Unitary (111) Orientation	1.38	1.27
Molar ratio of Ni to O_L	1.34 : 1	1.27 : 1	1.19 : 1
